# Emerging fungal pathogen *Ophidiomyces ophiodiicola* in wild European snakes

**DOI:** 10.1038/s41598-017-03352-1

**Published:** 2017-06-19

**Authors:** Lydia H. V. Franklinos, Jeffrey M. Lorch, Elizabeth Bohuski, Julia Rodriguez-Ramos Fernandez, Owen N. Wright, Liam Fitzpatrick, Silviu Petrovan, Chris Durrant, Chris Linton, Vojtech Baláž, Andrew A. Cunningham, Becki Lawson

**Affiliations:** 1 Institute of Zoology, Zoological Society of London, Regent’s Park, London, NW1 4RY UK; 2 US Geological Survey–National Wildlife Health Center, Madison, Wisconsin 53711 USA; 3 School of Biosciences, Cardiff University, Cardiff, CF10 3AX Wales UK; 4 Froglife, 1 Loxley, Peterborough, Cambridgeshire, PE4 5BW UK; 5 Public Health England, Myrtle Road, Bristol, BS2 8EL UK; 6 Department of Ecology and Diseases of Game, Fish and Bees, University of Veterinary and Pharmaceutical Sciences Brno, Brno, Czech Republic; 7 University College London, Gower Street, London, WC1E 6BT UK; 8 Conservation Science Group, Department of Zoology, University of Cambridge, The David Attenborough Building, Cambridgeshire, CB2 3QZ UK; 9 IDEXX Laboratories Limited, Grange House, Sandbeck Way, Wetherby, West Yorkshire LS22 7DN UK; 10 Naturemetrics, Unit 2 Littleton House, Littleton Road, Ashford, Surrey TW15 1UU UK

## Abstract

Snake fungal disease (SFD) is an emerging disease of conservation concern in eastern North America. *Ophidiomyces ophiodiicola*, the causative agent of SFD, has been isolated from over 30 species of wild snakes from six families in North America. Whilst *O. ophiodiicola* has been isolated from captive snakes outside North America, the pathogen has not been reported from wild snakes elsewhere. We screened 33 carcasses and 303 moulted skins from wild snakes collected from 2010–2016 in Great Britain and the Czech Republic for the presence of macroscopic skin lesions and *O. ophiodiicola*. The fungus was detected using real-time PCR in 26 (8.6%) specimens across the period of collection. Follow up culture and histopathologic analyses confirmed that both *O. ophiodiicola* and SFD occur in wild European snakes. Although skin lesions were mild in most cases, in some snakes they were severe and were considered likely to have contributed to mortality. Culture characterisations demonstrated that European isolates grew more slowly than those from the United States, and phylogenetic analyses indicated that isolates from European wild snakes reside in a clade distinct from the North American isolates examined. These genetic and phenotypic differences indicate that the European isolates represent novel strains of *O. ophiodiicola*. Further work is required to understand the individual and population level impact of this pathogen in Europe.

## Introduction

Fungal pathogens are emerging at an alarming rate worldwide and pose a significant threat to wildlife health^1^. Diseases, such as amphibian chytridiomycosis (caused by *Batrachochytrium dendrobatidis* and *B. salamandrivorans*) and white-nose syndrome of bats (caused by *Pseudogymnoascus destructans*), are causing dramatic biodiversity losses and are of great conservation concern^2–4^. In these instances, disease-induced population declines are believed to be the result of introduction of a novel pathogen into areas with naïve hosts.

Snake fungal disease (SFD), caused by the fungus *Ophidiomyces ophiodiicola* ^5^, is an emerging infectious disease of free-living wild snakes in North America that first gained widespread attention in 2008^6^ *. Ophidiomyces ophiodiicola* is widely distributed in eastern North America and is considered the main cause of fungal skin infections of wild snakes in that region^7–9^. The disease often causes mild infections^9^, but severe morbidity and mortality with consequent population declines have also been observed in wild snakes, including in some threatened species^6, 10^.

*Ophidiomyces ophiodiicola* is only known to infect snakes. It remains undetermined whether the fungus is an introduced pathogen or whether it is native to North America and currently emerging due to recent environmental change or other drivers^9^. *Ophidiomyces ophiodiicola* has been isolated from captive snakes with skin lesions from multiple continents since the 1980s, including Europe^11^: however, reports in wild snakes have been limited to eastern North America^9^. In this study, we present the first detections of *O. ophiodiicola* and SFD in wild snake species in Europe and compare European isolates of the fungus to those from the eastern United States.

## Results

### Detection of *Ophidiomyces ophiodiicola* in wild European snakes

A total of 336 wild snake samples from Great Britain (GB) (n = 33 carcasses and 302 moulted skins) and the Czech Republic (n = 1 moulted skin) were examined for macroscopic skin lesions consistent with *O. ophiodiicola* infection (Table 1)^5, 9^. Skin lesions were observed in 80 (23.8%) of the samples and these were screened for the presence of the fungus using real-time PCR. *Ophidiomyces ophiodiicola* was detected in 25 samples from snakes with macroscopic skin lesions, comprising 24 from British grass snakes (*Natrix natrix*) (eight carcasses and 16 moulted skins) and a moulted skin from a dice snake (*Natrix tessellata*) from the Czech Republic (Table 1). Panfungal PCR and DNA sequencing of the D1-D2 region of the large subunit of the ribosomal RNA gene of one of the PCR-positive grass snakes further corroborated the identity of the fungus as *O. ophiodiicola* (99% sequence identity to available *O. ophiodiicola* strains in GenBank; GenBank Accession LT607735). Dates of collection of the PCR-positive samples ranged from 2010–2016 with a wide geographical distribution in GB. The Czech Republic detection indicates that the fungus also occurs in mainland Europe (Supplementary Fig. S1). Swab samples from 11 grass snake carcasses and eight adder carcasses (*Vipera berus*) with no detected macroscopic lesions were similarly tested. A single snake, an adder, without macroscopic skin lesions was PCR-positive.

**Table 1:** The number of European snake species examined according to sample, if macroscopic lesions were detected and whether the samples were positive for *O. ophiodiicola* on real-time PCR.

Species	Sample	Number of samples examined	Number of samples with macroscopic skin lesions detected	Number of real-time PCR-positive samples
Grass snake (*Natrix natrix*)	Carcass	23	12	8
	Moulted skin shed	164	38	16
Adder (*Vipera berus*)	Carcass	10	2	1
	Moulted skin shed	107	20	0
Smooth snake (*Coronella austriaca*)	Moulted skin shed	31	7	0
Dice snake (*Natrix tessellata*)	Moulted skin shed	1	1	1
**Total**		**336**	**80**	**26**

Fungal culture conducted on skin lesions from 18 of the PCR-positive snakes led to the isolation of *O. ophiodiicola* from six of these animals (the dice snake and five grass snakes) (Supplementary Worksheet S1). In each case, the fungus was identified by PCR and sequencing of the internal transcribed spacer (ITS) region.

### Pathological investigations of *Ophidiomyces ophiodiicola* infections in European snakes

**Figure 1:**
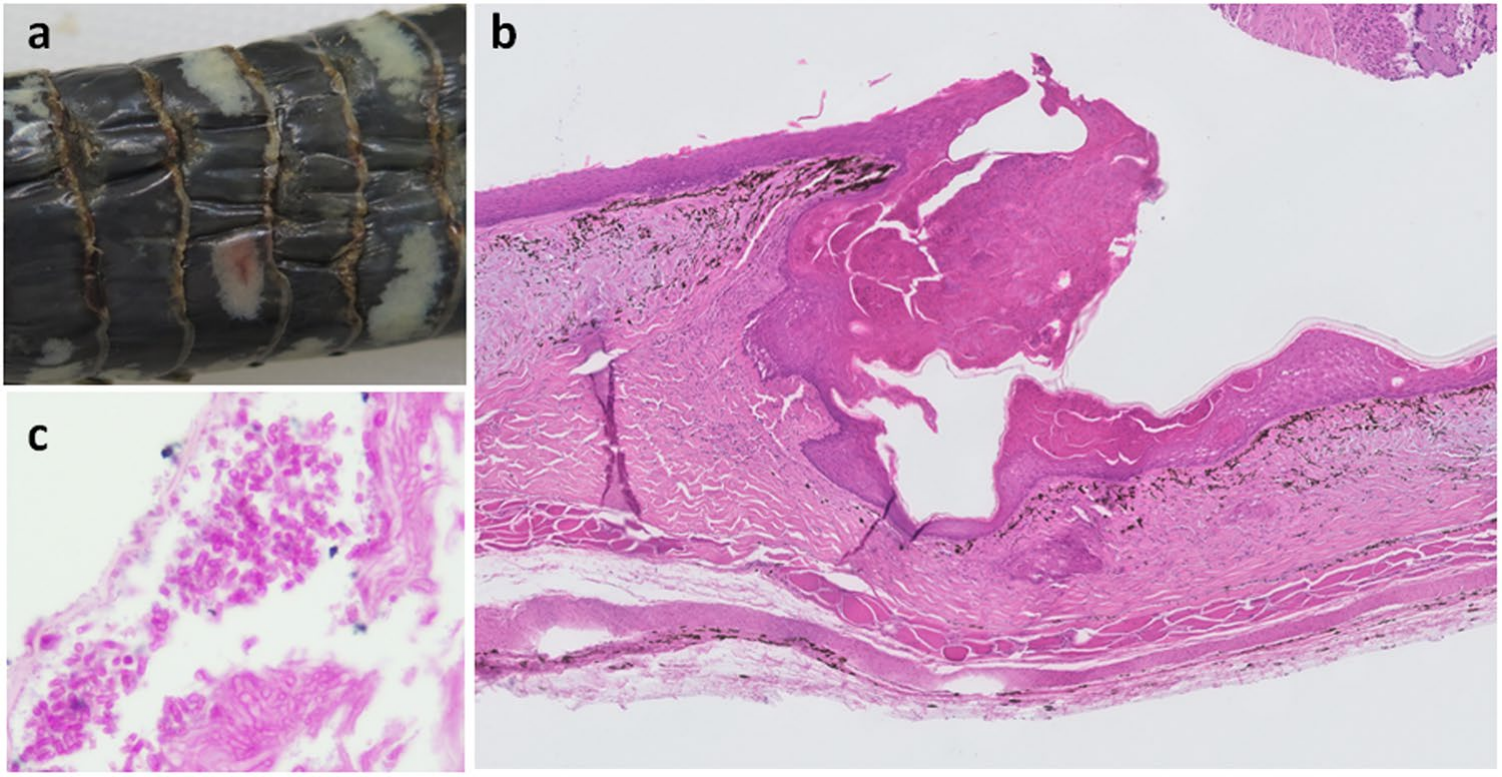
(**a**) Macroscopic lesions of snake fungal disease in a grass snake (*Natrix natrix*) showing thickened, yellow-brown areas mostly at the edges of the ventral scales with irregular margins (case: XT1041-16); (**b**) Microscopic lesions of snake fungal disease in a grass snake showing epidermal thickening and necrosis and dermatitis (case: X1041-16), HE stain, 50x magnification; (**c**) Focused area of microscopic lesion showing presence of arthroconidia in grass snake (case: XT804-15), PAS stain, 1000x magnification.

A systematic post-mortem examination and histopathology were conducted on eight of the European snakes with skin lesions from which suitable formalin-fixed tissues were available. Of these, five grass snakes from GB were positive for *O. ophiodiicola* on PCR examination (Supplementary Worksheet S2). Macroscopic lesions in these five cases had varied appearance, with multifocal areas of brown discoloration and thickening primarily affecting the ventral body scales (Fig. 1a). Lesions were observed particularly along the scale edges and in the crypts between affected scales, often with irregular caudal scale margins. Suspected dysecdysis (e.g., areas of flaking skin) was evident in two grass snakes. Microscopic examination (Fig. 1b) identified epidermal thickening and necrosis, of variable severity, with intralesional fungal hyphae (2–4 µm -diameter, parallel-walled, septate, branching) and mixed bacterial colonies (Fig. 1b). Arthroconidia with a size and morphology consistent with *O. ophiodiicola* were detected in three of the grass snakes with SFD (Fig. 1c). There was evidence of dermatitis in four snakes and associated myositis in two of these cases. Snake fungal disease was considered likely to be a direct or indirect cause of mortality in four of the five grass snakes with *O. ophiodiicola* infection: cachexia and dehydration were noted in three cases, one of which was euthanized for humane reasons, and predation in two cases. Accidental electrocution was considered the likely cause of death in the fifth grass snake with *O. ophiodiicola* infection. No other significant pathogens or concurrent significant infectious disease processes were detected in any of these snakes (Supplementary Worksheet S2).

Macroscopic lesions in the three grass snakes that were negative for *O. ophiodiicola* infection on PCR examination had a varied appearance (Supplementary Worksheet S2). Microscopic examination identified epidermal necrosis with intralesional bacteria in all three cases. Dermatitis of variable severity was present in two of these PCR-negative snakes. Fungal hyphae were observed infrequently in the skin of one case and suspected fungal hyphae were present in a second case but no arthroconidia were detected in any of these three snakes.

Lesions observed in all the moulted skin samples positive for *O. ophiodiicola* were mild, consisting of single to multifocal areas of brown discoloration and thickening affecting the ventral scales that ranged in size from 0.5 × 0.5 mm to 10 × 6 mm. Histopathology was not conducted on the moulted skins due to the nature of the samples.

### Characterisation of European isolates of *Ophidiomyces ophiodiicola*

**Figure 2:**
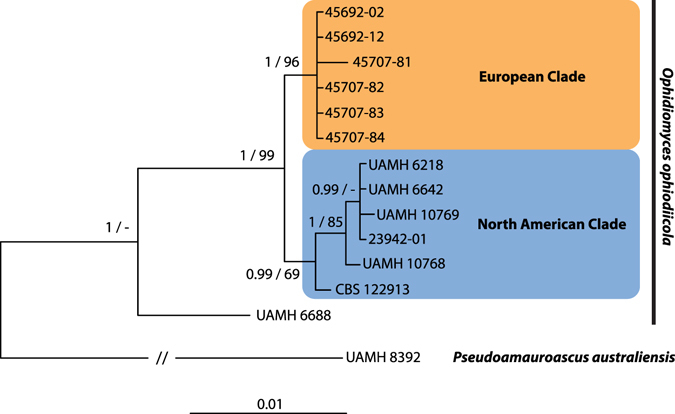
Phylogenetic tree resulting from Bayesian analysis of three concatenated loci (internal transcribed spacer region, and portions of the actin and translation elongation factor 1-α genes) of *Ophidiomyces ophiodiicola* isolates from eastern North America and Europe. The consensus tree resulting from a maximum likelihood analysis had the same topology. Posterior probabilities (Bayesian)/bootstrap support values (maximum likelihood) are displayed at nodes when the probabilities and bootstrap values were above 0.95 and 65, respectively. The tree is rooted with *Pseudoamauroascus australiensis*. Isolates of *O. ophiodiicola* from wild European snakes formed a clade distinct from isolates of the fungus from eastern North America. Isolate UAMH 6688 originated from captive snake at a zoological park in the United Kingdom in 1985 and is divergent from both clades.

To understand the relationship of European isolates of *O. ophiodiicola* with the fungus in North America, a phylogenetic analysis was performed using multiple chromosomal loci (ITS and partial sequences of the actin [ACT] and translation elongation factor 1-α [TEF] genes) from a subset of six European and six North American isolates (Supplementary Table S1). DNA sequences generated from *O. ophiodiicola* isolates cultured from wild snakes in GB were 100% identical to one another at all three loci; the Czech isolate differed from the GB isolates at each locus by one single nucleotide polymorphism (SNP; i.e., three SNPs over the region examined). The GB and Czech isolates formed a single, well-supported clade using both maximum likelihood and Bayesian analyses. This “European clade” was distinct from isolates of *O. ophiodiicola* that originated from snakes in eastern North America (Fig. 2). The isolates from both wild European snakes and North American snakes were genetically divergent from a strain of *O. ophiodiicola* previously isolated from a captive ball python (*Python regius*) in GB (UAMH Centre for Global Microfungal Biodiversity isolate number 6688;^10^).

During the process of culturing *O. ophiodiicola* from samples in this study, it was noted that the European isolates appeared to grow more slowly than those from snakes in eastern North America. To assess this quantitatively, experiments were conducted to compare the growth rates of the various *O. ophiodiicola* strains at 30 °C. The six isolates from wild European snakes had colony expansion rates that were, on average, 4.8 times slower than those previously isolated from the eastern United States (Supplementary Fig. S2). This phenotypic difference, along with their genetic distinctiveness, suggests that European isolates represent novel strains of *O. ophiodiicola* that differ from those associated with SFD emergence in North America.

## Discussion

We report the detection of *O. ophiodiicola* infection and SFD in wild European snakes. Previously, *O. ophiodiicola* had been detected in free-living snakes only in eastern North America^9^. Our sampling indicates that the pathogen is widely distributed in England and Wales, and is present in mainland Europe as evidenced by the occurrence of the fungus in the Czech Republic. Furthermore, the fungus has been present in Europe since at least 2010, but it might have occurred for much longer and previously had been overlooked.

Genetic and culture characterisations indicate that the European *O. ophiodiicola* isolates are distinct from the strains known to infect snakes in the eastern United States. Based on phylogenetic analyses, the isolates analysed from GB and the Czech Republic formed a well-supported “European clade” that was divergent from the clade containing American isolates. All of the isolates of *O. ophiodiicola* analysed from GB had identical DNA sequences in the loci examined, therefore it is possible that the fungus expanded clonally in GB from a point introduction. However, slight differences in DNA sequence data between the Czech and GB isolates demonstrate genetic diversity within Europe. Additional sampling and genetic characterisation is warranted to determine the extent of this diversity. Just as our study provides no current evidence to indicate that *O. ophiodiicola* was recently introduced to Europe, it similarly does not implicate Europe as a likely introduction source of the fungus to North America. We hypothesize that *O. ophiodiicola* naturally has a Holarctic distribution. Environmental change, such as habitat degradation and climate change, together with host factors such as inbreeding depression and underlying impaired health, might be implicated in the emergence of SFD^9, 10^.

*Ophidiomyces ophiodiicola* was detected in association with macroscopic skin lesions in 24 grass snakes and one dice snake. The fungus was also detected from one adder with no observed macroscopic lesions. The possibility that small skin lesions were present and missed on examination of this adder, however, cannot be excluded. Five of the snakes that were *O. ophiodiicola* PCR-positive had histologic lesions consistent with SFD with intralesional fungal organisms. Microscopic examination showed that infection was restricted to the skin, although two snakes also had localized myositis associated with skin lesions. Overall, *O. ophiodiicola* infection varied from mild to severe and was considered likely to be a direct or indirect cause of mortality in four of these animals. All moulted skins that were examined had relatively minor lesions: whilst it was not possible to determine the appearance of the skin lesions that these moult lesions corresponded to in live snakes, we consider these to be consistent with the mild, non-lethal infections reported in some North American snake populations^9^. The slower growth rate of European strains of *O. ophiodiicola* could influence pathogenicity and favour infections that are self-limiting. However, the presence of severe infections in some of the grass snakes examined in this study demonstrates that, as in North America, *O. ophiodiicola* can cause significant disease in European snakes^5, 9^.

Screening of moulted skins for macroscopic lesions and PCR testing may offer a practical means to conduct non-invasive surveillance for *O. ophiodiicola*. As demonstrated in this study, this can be achieved in a cost-effective manner by engaging herpetologists, ecologists and citizen scientists in the field. However, caution must be exercised with interpreting results obtained from moulted skins. Specifically, shed skin may have limited utility for confirming infection by histopathology, and shed skins could potentially harbour *O. ophiodiicola* originating from environmental sources rather than from the snake itself. Moulted skins found in the environment may persist for some time, and multiple shed skins found at a location might belong to a single snake. Thus, while moulted skins are a potential means by which to detect the presence of *O. ophiodiicola*, they are not suitable for characterising disease state, confirming aetiology, or determining the prevalence of infection within a population.

Establishing the range of *O. ophiodiicola* and historic occurrences of SFD outside of eastern North America is of great importance for understanding the ecology of this pathogen. Scanning surveillance of wild snakes should be prioritized to learn more about *O. ophiodiicola* and its ability to cause disease in different snake populations. This could initially occur through low cost efforts using minimally invasive techniques such as swabbing live snakes, screening macroscopic lesions from moulted skins and analyzing tissues from snakes undergoing post-mortem examinations. If *O. ophiodiicola* is detected during such surveillance or opportunistic sampling efforts, field studies in collaboration with experienced herpetologists may be justified to examine wild snakes for evidence of skin lesions consistent with SFD. More-invasive techniques, such as the collection of skin biopsy samples conducted under anaesthesia by veterinarians, could be used to confirm the diagnosis^9^. Biosecurity protocols to avoid anthropogenically-mediated pathogen spread and animal welfare considerations to minimise disturbance, should be incorporated into field studies. When possible, isolates of *O. ophiodiicola* should be obtained and archived to help address research questions related to origins of the fungus and potential differences in virulence between strains.

The individual and population level impacts of SFD in Europe remain unknown due to the challenges of reptile health surveillance and a paucity of long-term monitoring data^12^. Further research is required on the distribution, severity, host range, and epidemiology of *O. ophiodiicola* infection to determine the significance of SFD to the health of wild snake populations in Europe.

## Methods

### Sample collection

A total of 33 wild snake carcasses and 303 moulted wild snake skins that were collected from 2010–2016 were included in this study. These were submitted by members of the public and herpetologists as part of scanning disease surveillance and population genetics studies. All samples originated from GB with the exception of a single moulted skin from a dice snake found in the region of Brno in the Czech Republic (Supplementary Worksheet S1). Carcasses that could not be immediately examined or processed were stored frozen at −20 °C. Moulted skins were dried and stored at 4 °C. All samples were individually packaged and standard laboratory disinfection and decontamination procedures were followed to minimize the possibility of cross-contamination.

### Pathological investigations

Carcasses and moulted skins were examined for the presence of macroscopic skin lesions consistent with SFD^5, 9^. When present, a small portion of skin lesion (approximately 2mm × 2mm) was excised and stored at −80 °C until PCR and culture analyses could be performed. The skin of 19 snake carcasses without detected lesions was also swabbed to determine if snakes without apparent skin disease might harbour *O. ophiodiicola*. In some cases, lesions had been noted during the original post-mortem examination but samples were not taken and on re-examination of the frozen-thawed carcass, the lesions were no longer apparent. In these cases, a swab [MW113 peel pouch dry swab fine tip rayon swab; MWE Medical Wire, United Kingdom] moistened with sterile saline solution was passed along the whole length of the ventral body surface five times while rotating the swab slightly each time.

Saline mounts of small intestine contents were routinely examined microscopically for protozoan or metazoan parasites. Bacterial culture of the liver and small intestine contents was conducted on a subset of examinations using Columbia blood +5% horse blood agar and XLD plates incubated at 25 °C for 48 hours under aerobic conditions. A suite of tissue samples were taken for histopathological examination (Supplementary Worksheet S2), fixed in 10% neutral buffered formalin, embedded in paraffin and sectioned before they were stained with haematoxylin and eosin using routine methods. Sections of the skin lesions, and other tissues where indicated, were stained with Gram’s, Ziehl-Neelsen and Periodic acid-Schiff stains.

### Detection of *Ophidiomyces ophiodiicola*

DNA was extracted from swabs and skin tissue samples using PrepMan® Ultra Sample Preparation Reagent, (Applied Biosystems, Foster City, CA) with zirconium/silica beads^13^. Real-time *O. ophiodiicola*-specific PCR targeting the internal transcribed spacer (ITS) region was conducted using 5 µl of the extracted DNA as described by Bohuski *et al*.^14^. In addition, a standard panfungal PCR and DNA sequencing of the D1-D2 region of the large subunit of the ribosomal RNA gene^15^ was performed (Supplementary Table S2) using DNA extracted from a skin lesion of one of the snakes suspected to have SFD and which was positive for *O. ophiodiicola* using real-time PCR.

To confirm the PCR findings and obtain isolates for characterisation of European strains of *O. ophiodiicola*, fungal culture using dermatophyte test medium (DTM) as described by Lorch *et al*.^5^ was performed on skin lesions taken from a subset of 18 affected snakes. Fungal colonies that resembled *O. ophiodiicola* ^11^ were identified by sequencing the ITS region of the ribosomal RNA gene^16^ (Supplementary Worksheet S1).

### *Ophidiomyces ophiodiicola* strain characterisation

Pure cultures of six European isolates and five (colony expansion analysis) or seven (phylogenetic analyses) previously described isolates of *O. ophiodiicola* from eastern North America (Supplementary Table S1) were selected for further characterisation.

For phylogenetic analyses, DNA was isolated from fungal cultures using a phenol-chloroform extraction method, and PCR targeting portions of the ACT and TEF genes was conducted on the extracted DNA. Primers and cycling conditions for these reactions are presented in Supplementary Table S2. The resulting PCR products were gel purified (when necessary) and sequenced in both directions. Sequences of the ITS region (obtained as described above) and of the amplified portions of the ACT and TEF genes were aligned separately using ClustalW^17^ in MEGA version 6^18^; all gaps in the alignment were deleted. The 5.8S ribosomal RNA gene was removed from the ITS alignment because it was identical between all isolates examined. Model selection for each locus was performed in MEGA. The loci were then concatenated and the final alignment, consisting of 1,764 total characters (374 for ITS, 751 for ACT, and 639 for TEF), was used to conduct maximum likelihood and Bayesian analyses using the CIPRES Science Gateway^19^. For both analyses, the loci were partitioned, with an HKY85 nucleotide substitution model applied to the ITS data, and a Kimura 2-parameter model applied to the ACT and TEF sequence data. Maximum likelihood analysis was performed in PAUP* version 4.0a150^20^ with 1,000 bootstrap replicates and heuristic search to find the best tree topology. Bayesian analysis was conducted using MrBayes version 3.2.6^21^ with two runs, four chains, and 5,000,000 generations with the first 25% used as burnin and a sampling frequency of 1,000 generations. The trees were rooted with *Pseudoamauroascus australiensis*.

Growth rates were measured by stab-inoculating DTM plates at three separate locations using a pre-sterilized metal pick touched to an actively growing culture; plates were inoculated in duplicate such that there were a total of six colonies per isolate. The newly inoculated cultures were incubated at 30 °C, and colony diameter was measured to the nearest 0.5 mm at days 6 and 15. The change in diameter over this nine day period was converted to change in area (using the equation for calculating area of a circle) for each colony.

## Electronic supplementary material

Supplementary Information

Supplementary Datasheet S1

Supplementary Datasheet S2
