# Supplementary material for: Emerging fungal pathogen Ophidiomyces ophiodiicola in wild European snakes
Source: Sci Rep. 2017 Jun 19;7:3844. doi: 10.1038/s41598-017-03352-1 (PMC5476567; doi:10.1038/s41598-017-03352-1)
Supplement: Supplementary file 1 — Supplementary Information [file 41598_2017_3352_MOESM1_ESM.pdf]

Emerging fungal pathogen *Ophidiomyces ophiodiicola* in wild European snakes

Lydia H. V. Franklinos, Jeffrey M. Lorch, Elizabeth Bohuski, Julia Rodriguez-Ramos Fernandez, Owen N. Wright, Liam Fitzpatrick, Silviu Petrovan, Chris Durrant, Chris Linton, Vojtech Baláž, Andrew A. Cunningham, Becki Lawson

SUPPLEMENTARY MATERIALS

Supplementary Figure S1: (a) Distribution of *Ophidiomyces ophiodiicola* PCR-positive snakes in Great Britain, 2010-2016, superscript denotes the number of positive samples when n>1. (b) Distribution of *Ophidiomyces ophiodiicola* PCR-positive snakes in Europe, 2010-2016. Species comprised grass snakes (*Natrix natrix*) and adder (*Vipera berus*) in Great Britain and dice snake (*Natrix tessellata*) in the Czech Republic.

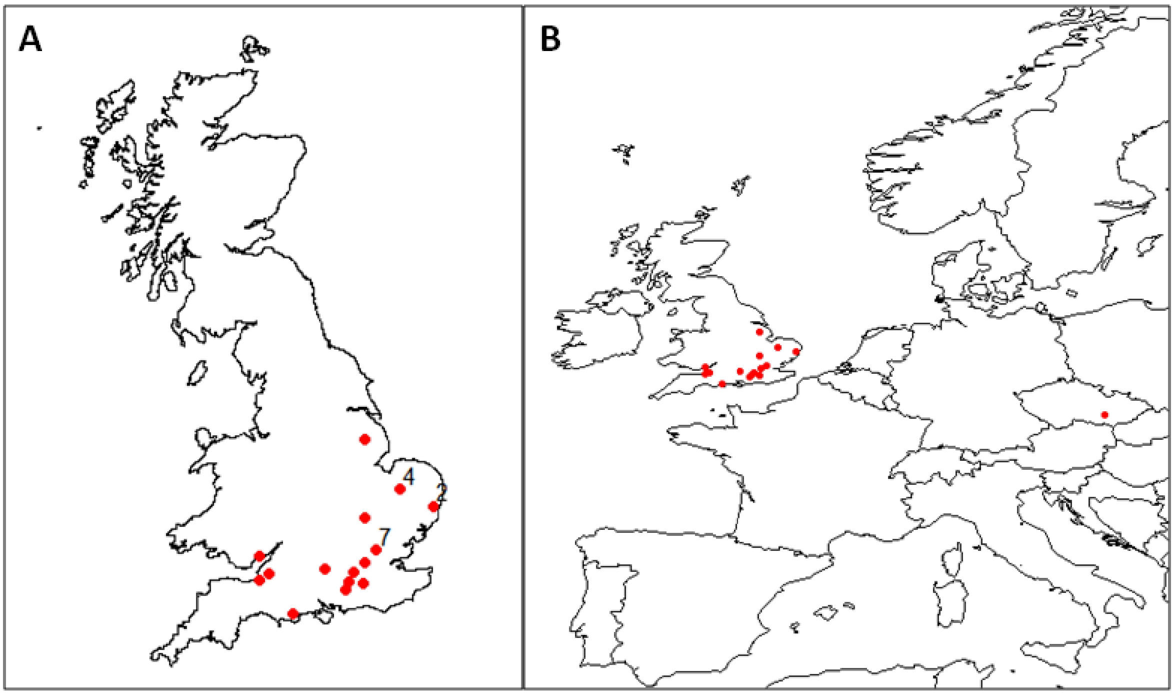

Maps were generated by O.N.W. using R version 3.3.2 (2016-10-31) available to download at https://cran.r-project.org/bin/windows/base/ and the following packages 'maps', 'mapdata', 'calibrate' and 'rworldmap'.

R core team. *R: A language and environment for statistical computing*. (R Foundation for Statistical Computing, 2016).

Supplementary Figure S2: Growth rates of various *Ophidiomyces ophiodiicola* strains. Changes in colony area were measured for a nine day growth period (from days 6-15 post-inoculation) at 30 °C. Strains represented with blue bars originated from snakes in the eastern United States, whereas strains isolated from wild European snakes are shown in yellow. Errors bars depict standard deviation. Pictures of fungal colony size at 12 days post-inoculation for representative American (CBS 122913; upper) and European (NWHC 45707-83; lower) isolates are also shown.

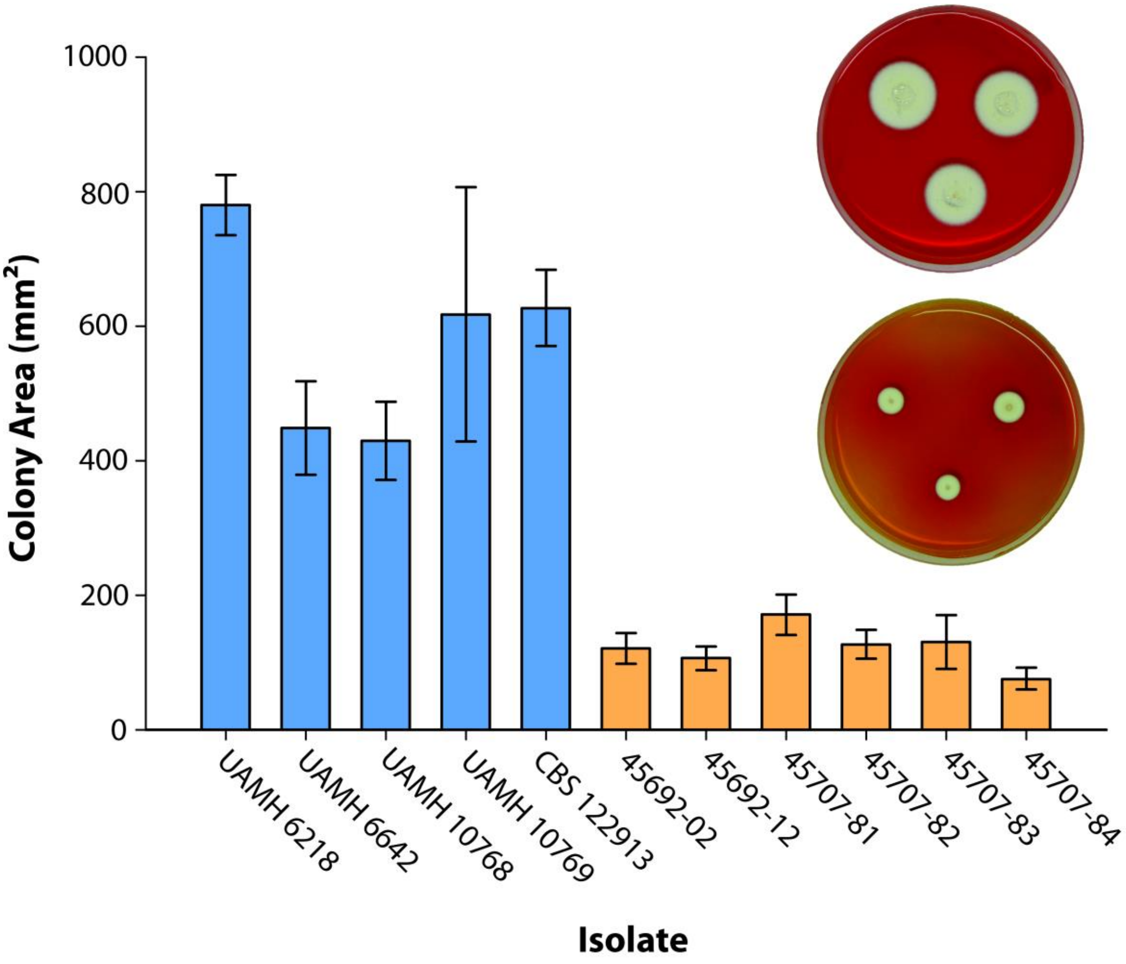

Supplementary Table S1: List of *Ophidiomyces ophiodiicola* isolates used in the growth rate and phylogenetic analyses. GenBank accession numbers for loci used in the phylogenetic analysis are provided.

Isolate Number	Fungal Species	Location	Analysis	Genbank accession numbers		
				ITS	ACT	TEF
UAMH 6218	*Ophidiomyces ophiodiicola*	New York, USA	phylogenetic / growth	KF477227	KY474066	KY474080
UAMH 6642	*Ophidiomyces ophiodiicola*	Maryland, USA	phylogenetic / growth	KC884267	KY474067	KY474081
UAMH 6688	*Ophidiomyces ophiodiicola*	UK	phylogenetic	KF477228	KY474078	KY474082
UAMH 10768	*Ophidiomyces ophiodiicola*	Florida, USA	phylogenetic / growth	KF477234	KY474068	KY474083
UAMH 10769	*Ophidiomyces ophiodiicola*	Florida, USA	phylogenetic / growth	KF477235	KY474069	KY474084
CBS 122913	*Ophidiomyces ophiodiicola*	Georgia, USA	phylogenetic / growth	EU715819	HF547891	KY474079
NWHC 23942-01 / ATCC MYA-4974	*Ophidiomyces ophiodiicola*	Massachusetts, USA	phylogenetic	KY474065	KY474076	KY474091
NWHC 45692-02	*Ophidiomyces ophiodiicola*	Surrey England (GB)	phylogenetic / growth	KY474059	KY474070	KY474085
NWHC 45692-12	*Ophidiomyces ophiodiicola*	Suffolk England (GB)	phylogenetic / growth	KY474060	KY474071	KY474086
NWHC 45707-81	*Ophidiomyces ophiodiicola*	South Moravia (Czech Republic)	phylogenetic / growth	KY474061	KY474072	KY474087
NWHC 45707-82	*Ophidiomyces ophiodiicola*	Suffolk England (GB)	phylogenetic / growth	KY474062	KY474073	KY474088
NWHC 45707-83	*Ophidiomyces ophiodiicola*	Somerset England (GB)	phylogenetic / growth	KY474063	KY474074	KY474089
NWHC 45707-84	*Ophidiomyces ophiodiicola*	Greater London England (GB)	phylogenetic / growth	KY474064	KY474075	KY474090
UAMH 8392	*Pseudoamauroascus australiensis*	Mount Lofty, Australia	phylogenetic	AJ131787	KY474077	KY474092

ATCC = American Type Culture Collection

CBS = Centraalbureau voor Schimmelcultures

NWHC = U.S. Geological Survey - National Wildlife Health Center

UAMH = UAMH Centre for Global Microfungal Biodiversity

Supplementary Table S2: Polymerase chain reaction primers and cycling conditions used in this study.

Locus	Primers	Cycling Conditions	Reference
D1-D2	LSU266F:<br>5' -GAGTCGAGTTGTTTGGGAATGC - 3'<br>LSU654R:<br>5' - GGTCCGTGTTTCAAGACGG - 3'	94°C for 15 min; 40 cycles of 94°C for 15 sec, 65°C for 30 sec, 72°C for 1 min; final extension of 72°C for 7 min	Borman, A.M., Campbell, C.K., Linton, C.J., Bridge & P.D., Johnson, E.M. *Polycytella hominis* is a mutated form of *Scedosporium apiospermum*. *Med. Mycol.* **44**, 33–39 (2006).
ITS	ITS5:<br>5' - GGA AGT AAA AGT CGT AAC AAG G - 3'<br>ITS4:<br>5' - TCC TCC GCT TAT TGA TAT GC - 3'	98°C for 2 min; 40 cycles of 98°C for 10 sec, 50°C for 30 sec, 72°C for 1 min; final extension of 72°C for 7 min	White, T.J., Bruns, T., Lee, S. & Taylor, J.W. Amplification and direct sequencing of fungal ribosomal RNA genes for phylogenetics. pp. 315-322 in Innis, M.A., Gelfand, D.H., Sninsky, J.J. & White, T.J. (eds), PCR Protocols: A Guide to Methods and Applications. Academic Press Inc., New York. (1990).
ACT	ACT-1-CO:<br>5' - GAC GAT TAT GGA GAA GAT CTG GC - 3'<br>ACT-4r-CO:<br>5' - CCR ATC CAG ACV GAG TAT TTA CG - 3'	95°C for 5 min; 46 cycles of 94°C for 45 sec, 58°C for 45 sec, 72°C for 1 min; final extension of 72°C for 10 min	This study.
TEF	EF-DermF:<br>5' - CAC ATT AAC TTG GTC GTT ATC G - 3'<br>EF-DermR:<br>5' - CAT CCT TGG AGA TAC CAG C - 3'	96°C for 6 min; 41 cycles of 94°C for30 sec, 58°C for 30 sec, 72°C for 1 min; final extension of 72°C for 10 min	Mirhendi, H. *et al.* Translation elongation factor 1-α gene as a potential taxonomic and identification marker in dermatophytes. *Med. Mycol*. **53**, 215-224 (2015).
